# Infestation of introduced raccoons (*Procyon lotor*) with indigenous ixodid ticks on the Miura Peninsula, Kanagawa Prefecture, Japan

**DOI:** 10.1016/j.ijppaw.2018.09.002

**Published:** 2018-09-21

**Authors:** Kandai Doi, Takuya Kato, Shin-ichi Hayama

**Affiliations:** Nippon Veterinary and Life Science University, Japan

**Keywords:** Invasive alien species, *Haemaphysalis flava*, Intensity of infestation, *Procyon lotor*, Japan

## Abstract

Since the raccoon (*Procyon lotor*) was introduced to Japan, studies have established that they are infested with native Japanese tick species. However, the quantity of ticks infesting raccoons is unknown. We conducted a survey of ticks on invasive raccoons captured on the Miura Peninsula, Kanagawa Prefecture, Japan, from April 2015 through June 2016 to determine the species of ticks and to quantify the intensity of tick infestation in order to obtain basal information related to the ecology of host–parasite relationships among indigenous tick species and an alien mammalian species. We collected and identified 15,931 ticks of two genera and six species, namely, *Haemaphysalis flava*, *H. megaspinosa*, *H. longicornis*, *H. japonica*, *Ixodes ovatus*, and *I. tanuki*, from 100 out of 115 raccoons. The dominant tick species was *H. flava* (96.8%) and individuals were mainly adults. Seasonal patterns of infestation intensity of adults and nymphs peaked in the autumn and winter and decreasing in the late spring and summer, May to August, while larvae peaked in August. Our results indicated that host–parasite relationships between invasive raccoons and Japanese tick species, especially *H. flava*, were established in Kanagawa Prefecture. The ticks infest invasive raccoons for their blood-meal and also for overwintering. The results of this study extend our understanding of the ecology of tick-borne diseases.

## Highlights

• All developmental stages of *Haemaphysalis flava* infested feral raccoons.
• Adults *H. flava* was dominant tick infested raccoons.
• The peak of tick infestation raccoons was in late autumn and winter.
• Adults and nymphal *H. flava* use raccoons for blood-meal and overwintering.

## Graphical abstract

**Figure:**
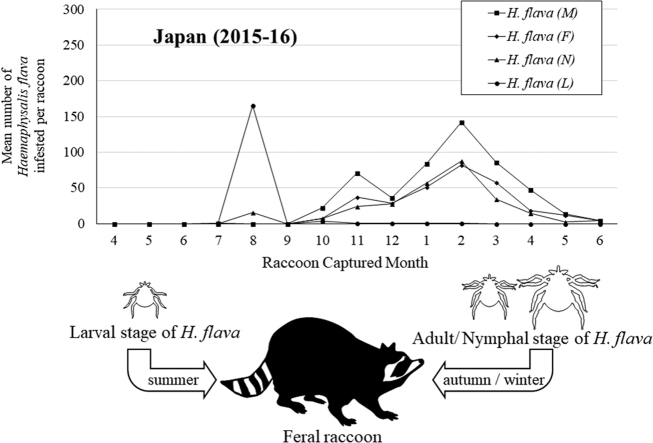

## 1 Introduction

The first report of feral raccoons (*Procyon lotor*) in Japan was reported in 1962 in Aichi Prefecture and the raccoon was imported to Japan as a pet and increased number in the late 1970s (Ikeda, 2006). A survey performed by the Japanese government in 2006 confirmed that raccoons have established populations throughout Japan (Ikeda, 2006). They compete for resources with native mammals, such as the raccoon dog (*Nyctereutes procyonoides*) and the red fox (*Vulpes vulpes japonica*; Ikeda, 2006; Ikeda et al., 2004), prey on endangered reptiles (Kaneda and Kato, 2011), damage crops (Ikeda, 2006), break into houses (Ikeda, 1999, 2006) and are recognized as an invasive species (Ikeda, 1999). Furthermore, they are hosts to ticks and associated pathogens that impact the health of human, livestock, and other indigenous wildlife. Multiple reports indicate that raccoons have a role as a reservoir of tick-borne diseases (TBD), (e.g., Japanese spotted fever; JSF) (Inoue et al., 2011), tularemia (Berrada et al., 2006; Fujita, 2009a; Inoue et al., 2011), babesiosis (Kawabuchi et al., 2005), and severe fever and thrombocytopenia syndrome (SFTS) (Takahashi et al., 2014). Since both human infestation and TBD case reports, such as JSF, concentrate in seasons, ticks are mostly active in the environment (Mahara, 1997). Also, TBD pathogens circulate between wildlife and ticks and the distribution of host wildlife is overlapped with vector ticks (Tsukada et al., 2014). Thus, the seasonal patterns of ticks and distributional patterns of ticks and host wildlife are strongly interacting each other. Moreover, on-host ticks observation indicates how vector ticks widen their distribution. However, the seasonal patterns of on-host ticks are not fully understood especially among introduced wildlife. and *Haemaphysalis flava*, *H. megaspinosa*, *H. longicornis*, *Amblyomma testudinarium*, *Ixodes nipponenis*, *I. tanuki*, and *I. ovatus* infest invasive raccoons in Japan (Fujita, 2009b; Kobayashi et al., 2012). These ticks contribute to the risk of transmitting TBD pathogens to raccoons (Furuno et al., 2017). The possibility that indigenous tick species and invasive raccoon developed a new host–parasite relationship in recent decades implies that a new route of TBD transmission has been developed by vector ticks transmitting TBD pathogens from native reservoir animals to a new host species (Fujita, 2009b). The feral raccoons in Kanagawa Prefecture, Japan may have resulted in changes of host availability for local ticks. These ticks may have experienced a rapid expansion of newly available hosts, while original hosts in the sympatric niche, such as raccoon dogs and red foxes, were driven away from the habitat (Ikeda, 1999; Ikeda et al., 2004). However, the details of the host–parasite relationships of raccoons and ticks in Kanagawa Prefecture are not well known, despite the recommendation that monitoring wildlife hosts and vector ticks are important to define the ecoepidemiology of TBD (Alexander et al., 2012).

The purpose of our research was to identify and quantify the species of ticks infesting raccoons. This survey is a first step to obtaining a full understanding the host–parasite relationship between invasive raccoons and Japanese ticks.

## 2 Material and methods

### 2.1 Study area

**Fig. 1:**
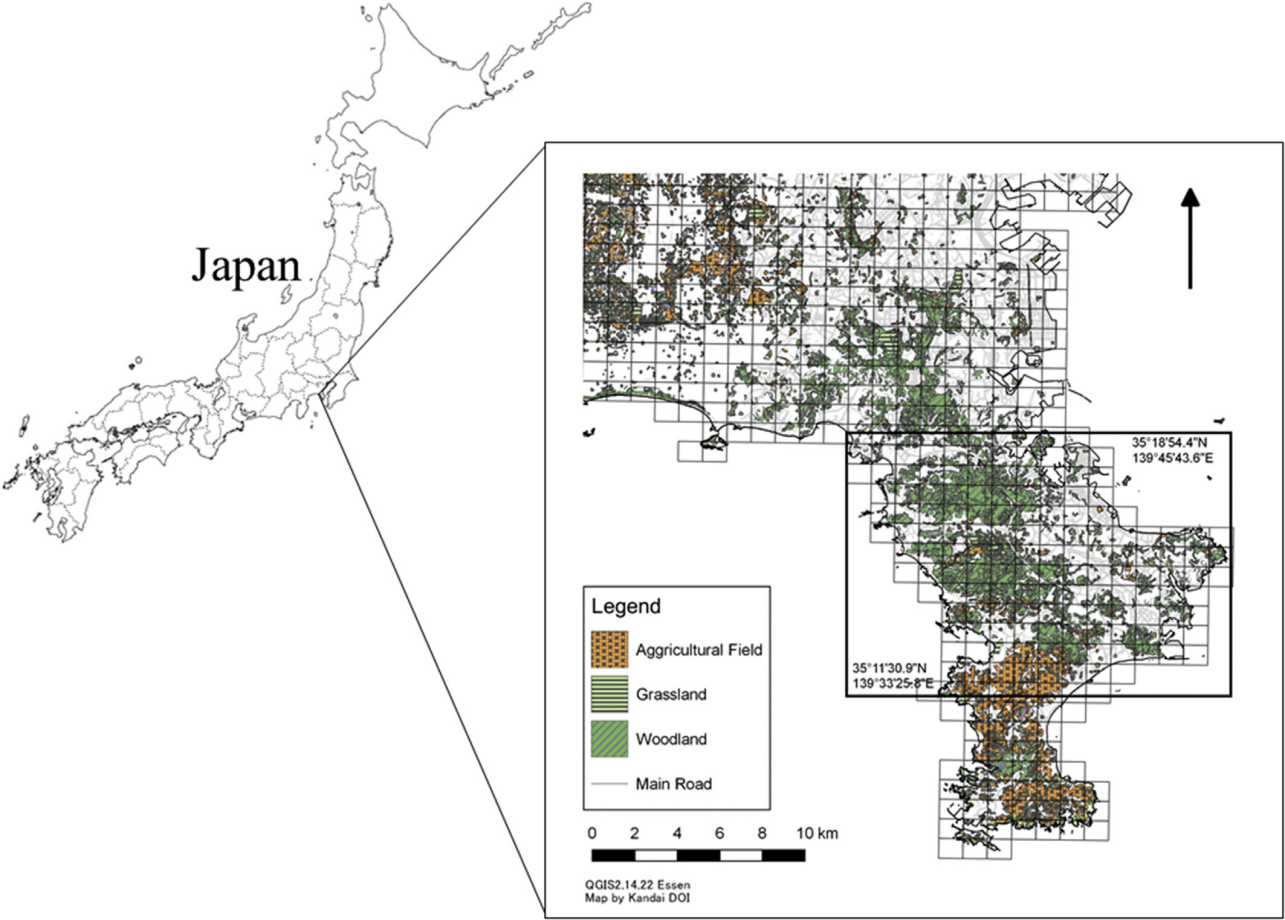
Map of the Miura Peninsula, Kanagawa Prefecture, Japan. The prefecture is located southeast of Kanto Plain. The square indicates our study area, which includes Yokosuka City and Hayama Town.

The study was conducted in Yokosuka City (35°16′53.5″N 139°40′19.4″E) and the town of Hayama (35°16′19.6″N 139°35′10.3″E), Miura Peninsula, Kanagawa Prefecture (Fig. 1). The area is located in the central eastern region of Honshu Island, Japan. The peninsula is characterized by a humid subtropical climate, classified as “humid subtropical climate” under the Köppen climate classification with four seasons: spring (March–May), summer (June–August), autumn (September–November), and winter (December–February) (Japan Meteorological Agency, 2016; Kottek et al., 2006).

Fauna in the study area included raccoon dog, Japanese badger (*Meles anakuma*), and invasive raccoons. The Japanese Sika deer (*Cervus nippon*), Japanese macaque (*Macaca fuscata*), and Japanese wild boar (*Sus scrofa leucomystax*) were not present based on an animal survey conducted by the Biodiversity Center of Ministry of the Environment of Japan (2004).

### 2.2 Raccoon samples

Since the Invasive Alien Species Act in 2005 listed the raccoon as an invasive alien species, local governments of Japan have implemented countermeasures against the biological and economical damage caused by raccoons (Ama, 2007). Kanagawa Prefecture initiated the Kanagawa Prefecture Raccoon Control Implementation Plan (2016) in 2006, where licensed hunters captured invasive raccoons using box traps (Havahart Model 1089; Woodstream, Pennsylvania, USA). Raccoons were euthanized with carbon dioxide gas following the Guidelines for Management of Invasive Alien Species of the Japan Veterinary Medical Association (Japan Veterinary Medical Association, 2007). During this study, raccoons were captured and euthanized from April 2015 through June 2016.

### 2.3 Tick collection and identification

Ticks were collected from 115 raccoon carcasses using forceps and combing all skin surfaces using a cat flea comb. Ticks were preserved in 70% ethanol and then identified to species, stages of development, and sex of adults based on morphological examination under a stereo-microscope and a biological microscope using standard keys (Yamaguti et al., 1971).

### 2.4 Data analysis

The mean burden and proportion of each tick species were estimated. The dominant species and seasonal abundance of ticks were determined.

## 3 Results

### 3.1 General findings

A total of 100/115 (87.0%) of the raccoons examined were infested with a total of 15,931 ticks belonging to six species and two genera (Table 1). The mean number of ticks collected per raccoon was 138.5 (range: 0–1346). A total of 55 ticks were not identified to species due to damages. *Haemaphysalis. flava* accounted for the highest number of ticks (15414; 96.8%), followed by *H. megaspinosa* (232; 1.5%), *I. ovatus* (98; 0.6%), *H. longicornis* (92; 0.6%), *I. tanuki* (38; 0.2%), and *H. japonica* (2; <0.1%). All stages of *H. flava*, *H. megaspinosa*, and *H. longicornis* were detected on raccoons. Raccoons were infested by adult males and females of *I. tanuki*, females only of *I. ovatus*, and only single individuals of a nymph and adult male *H. japonica* were collected (Table 1).

**Table 1:** Life stage* and sex of ticks infesting the raccoons captured in Kanagawa Prefecture, Japan from April 2015 through June 2016. *H. flava* was the most abundant species found on the raccoons. * M: male adult, F: female adult, N: nymph, L: larva.

Month (Number of raccoons infested)	*H. flava*				*H. megaspinosa*				*H. longicornis*				*H. japonica*				*I. tanuki*				*I. ovatus*				Unknown			
	M	F	N	L	M	F	N	L	M	F	N	L	M	F	N	L	M	F	N	L	M	F	N	L	M	F	N	L
2015 Apr (0/1)	Not Detected																											
2015 May (0/1)	Not Detected																											
2015 Jun (0/3)	Not Detected																											
2015 Jul (2/2)	2	0	0	0	0	0	0	0	0	0	0	0	0	0	0	0	0	0	0	0	0	0	0	0	0	0	0	0
2015 Aug (2/2)	0	0	32	331	0	0	0	0	0	0	0	0	0	0	0	0	0	0	0	0	0	0	0	0	0	0	0	0
2015 Sep (0/1)	Not Detected																											
2015 Oct (12/12)	267	83	81	43	3	14	0	3	1	0	0	1	0	0	0	0	0	0	0	0	0	0	0	0	0	0	0	4
2015 Nov (6/7)	496	259	168	2	2	5	3	0	0	1	1	0	0	0	0	0	0	0	0	0	0	0	0	0	0	0	0	0
2015 Dec (4/7)	256	198	191	4	10	14	3	0	0	0	0	0	0	0	0	0	0	0	0	0	0	0	0	0	0	0	8	0
2016 Jan (23/23)	1931	1169	1306	4	42	43	17	0	1	25	0	0	1	0	0	0	0	12	2	0	1	0	1	0	0	0	34	0
2016 Feb (17/18)	2554	1485	1584	5	5	7	6	0	0	4	0	0	0	0	0	0	0	10	2	0	1	13	2	0	0	0	1	0
2016 Mar (12/13)	1119	749	441	1	8	33	5	0	0	4	0	0	0	0	1	0	1	11	0	0	9	26	2	0	0	0	6	0
2016 Apr (4/4)	191	75	59	0	2	0	0	0	0	1	2	0	0	0	0	0	0	0	0	0	2	11	0	0	0	0	0	0
2016 May (4/4)	53	48	9	0	0	0	0	0	0	7	5	0	0	0	0	0	0	0	0	0	2	7	0	0	0	0	0	0
2016 Jun (14/17)	69	73	75	1	1	1	5	0	0	12	27	0	0	0	0	0	0	0	0	0	5	16	0	0	0	2	0	0
Total	6938	4139	3946	391	73	117	39	3	2	54	35	1	1	0	1	0	1	33	4	0	20	73	5	0	0	2	49	4
	15,414				232				92				2				38				98				55			

### 3.2 Seasonal patterns

**Fig. 2:**
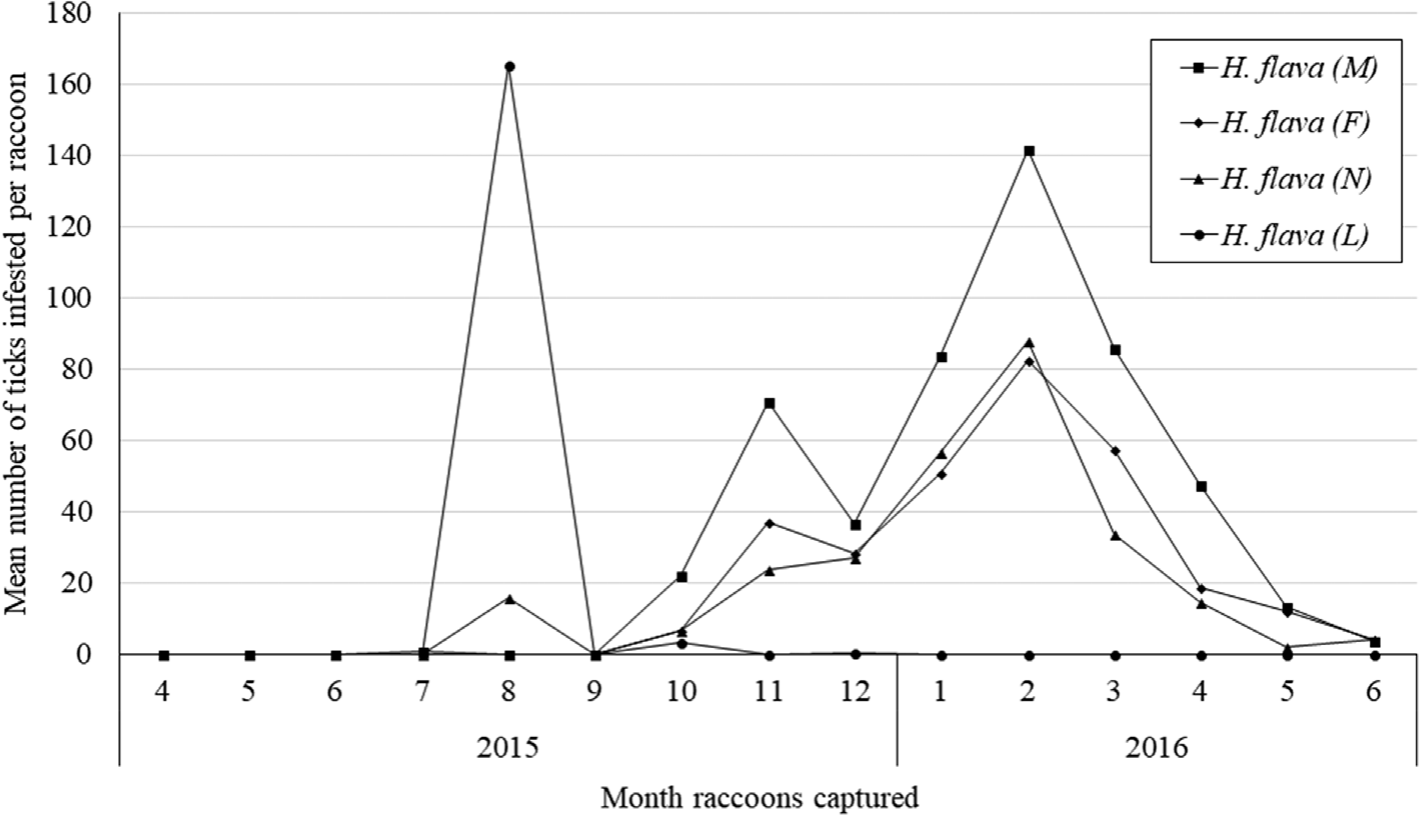
Temporal change of raccoon infesting *Haemaphysalis flava* in the Miura Peninsula, Japan from April 2015 to June 2016.

Temporal analyses of *H. flava* on the invasive raccoons demonstrated a relatively intense infestation in the autumn and winter which decreased in the late spring and summer, May to August (Fig. 2; Table 1). Adult ticks peaked in the winter, while larvae peaked in August. In addition, despite seasonal changes, *H. flava* used raccoons as a host throughout the year for every developmental stage (Table 1).

## 4 Discussion

Our results and previous studies which reported tick infestation on raccoons in Japan (Fujita, 2009b; Kobayashi et al., 2012) indicated that invasive raccoons and the Japanese native tick, *H. flava,* have a well established host-parasite relationship.

The previous study in Chiba Prefecture, Japan, about 80 km east of our study area, also reported that larval *H. flava* were the most abundant in the summer and adult *H. flava* were the most abundant in the autumn and winter (Asanuma, 1956; Saito, 1959). In addition, two previous tick surveys in the western region of the Republic of Korea during April and October by Cobrun et al. (2016), and in the southeastern region of the Republic of Korea during March and October by Johnson et al. (2017), reported that adult and nymphal *H. flava* were frequently collected in April, May and October, and decreased during August, while larvae were collected starting July and peaked in August. Moreover, Johnson et al. (2017) reported that the habitat preference of *H. flava* was the forest environment.

Our results indicated a relatively high intensity of adult and nymphal *H. flava* infestation that started to increase in November and peaked in February while larval *H. flava* peaked in August (Fig. 2). Asanuma and Sakurai (1958), Yoshida (1980), Fujimoto et al. (1987), and Kakuda et al. (1990) reported larval *H. flava* and *H. longicornis* overwintered on the host. Thus, our results of intense infestation on raccoons during winter indicated on-host overwinter habitat of adult and nymphal *H. flava*. *H. flava* is known that they frequently infest domestic dogs (*Canis lupus familiaris*) going into forest environment (Choe et al., 2011). Also, surveys in previous studies were conducted before invasive raccoons spread across Japan, infestations of *H. flava* (Saito, 1959; Saito et al., 1965), *H. longicornis* (Saito et al., 1965), *I. tanuki* (Saito, 1964), and *I. ovatus* (Saito, 1959) were found from medium-sized carnivores, including raccoon dogs, red foxes, Japanese weasels (*Mustela sibirica itatsi*), and Japanese badgers. These ticks established host–parasite relationships with indigenous medium-sized carnivores in the Miura Peninsula before or at the time of the introduction of raccoons. In addition, our study area, Miura Peninsula was the area that Japanese Sika deer and Japanese wild boar have not been observed in recent decades, which were known to be hosts of various tick species, such as *H. longicornis, H. megaspinosa, A. testudinarium,* and *I. ovatus*, (Biodiversity Center of Ministry of the Environment of Japan, 2005; Yamaguti et al., 1971; Tsukada et al., 2014). This implies that *H. flava*, *H. longicornis*, *I. ovatus*, and *I. tanuki* may have selected raccoons or at least used raccoons as hosts in addition to indigenous medium-sized carnivores because of its availability as the host. Although, Yamaguti et al. (1971) reported that larval *H. flava* prefer small mammals and small birds, we observed larvae *H. flava* infestation in August (Fig. 2). This indicated larval *H. flava* may be able to infest larger mammals (e.g., raccoon) if they were more abundant in the area or used the environment of the area frequently.

## 5 Conclusion

The relationships between native ticks and invasive raccoons in Kanagawa Prefecture, Japan was observed. Various tick species in Japan exhibited a high adaptability of infesting the invasive raccoons in our study area that were recently introduced in the past few decades. Every developmental stage of *Haemaphysalis flava* using raccoons as host for their blood-meal and especially adults and nymphal *H. flava* may have used raccoons for overwintering.

*H. flava*, *H. longicornis*, *H. megaspinosa*, *H. kitaokai*, *Amblyomma testudinarium* were known as vector ticks of SFTS. Also, *H. flava*, *H. longicornis*, *H. megaspinosa*, *H. japonica* and *I. ovatus* were the ticks that detections of the spotted fever group rickettsiae (Ishikura et al., 2000; Katayama et al., 1996, 2001) and cases of human infestation (Seishima et al., 2000; Yamauchi et al., 2010) were reported in the past. Raccoon, which was infested by ticks in our study area, is known as an urban wildlife breaking into houses and using various environment (e.g. forest, grassland, urban) (Ikeda, 2009). Thus, it is highly possible that ticks spread its distribution toward human living environment by infesting a raccoon and those ticks possibly harbor TBDs. It is necessary to perform tick surveys of medium-sized carnivores living in our study area to have a better understanding of the host preferences of various tick species and their associated pathogens they harbor in Japan.

## Conflicts of interest

None.
